# Effect of anesthesia strategy during endovascular therapy on 90-day outcomes in acute basilar artery occlusion: a retrospective observational study

**DOI:** 10.1186/s12883-020-01979-8

**Published:** 2020-10-29

**Authors:** Haibin Du, Xu Tong, Xuan Sun, Zhiyong Shi, Bin Liu, Feng Gao, Zhongrong Miao, Dong Zhang

**Affiliations:** 1 Department of Neurosurgery, Beijing Tiantan Hospital, Capital Medical University, No.119 South 4th Ring West Road, Fengtai District, Beijing, 100070 China; 2 Department of Neurosurgery, Peking University Shougang Hospital, Beijing, China; 3 Department of Interventional Neuroradiology, Beijing Tiantan Hospital, Capital Medical University, No.119 South 4th Ring West Road, Fengtai District, Beijing, 100070 China; 4 Department of Neurology, Tangshan Gongren Hospital, Hebei Medical University, Tangshan, Hebei China; 5 Department of Anesthesiology, Beijing Tiantan Hospital, Capital Medical University, Beijing, China

**Keywords:** Anesthesia, Basilar artery occlusion, Endovascular therapy, Outcome

## Abstract

**Background and objective:**

The research on the effect of anesthesia on endovascular therapy (EVT) of acute ischemic stroke is mainly focused on the anterior circulation, and little is known about the data of basilar artery occlusion (BAO). This study aims to investigate the association of anesthesia strategy with 90-day clinical outcomes of patients with acute BAO treated with EVT.

**Methods:**

We reviewed our prospectively collected data from the endovascular treatment database at the Beijing Tiantan Hospital. This included patients with acute BAO who had a documented 90-day modified Rankin Scale (mRS) score from January 2012 to July 2018. Options for EVT included general anesthesia (GA) and conscious sedation (CS) performed by an anesthesia care team in the institution. The recommendation of anesthesia for patients was a joint decision between anesthesiologist and neurointerventionalist according to a pre-designed scheme. Patients who required tracheal intubation for airway protection prior to EVT were excluded. The clinical outcomes we observed were functional independence (mRS ≤2), favorable outcome (mRS ≤3), and mortality at 90 days after the procedure. Univariate and multivariable logistic regression analyses were used to explore the relationship between anesthesia methods and 90-day outcomes.

**Results:**

A total of 187 patients with BAO were treated by EVT in this study. Nine cases requiring emergent intubation prior to EVT were excluded. 139 patients (78.1%) underwent GA and 39 patients (21.9%) underwent CS. In univariate analysis, GA was associated with less functional independence [odds ratio (OR), 0.28; 95% confidence interval (CI), 0.13–0.59] and less favorable outcome (OR, 0.23; 95% CI, 0.10–0.52) than was CS. After adjusting for potential confounders, multivariable analysis showed that there were still significant differences between GA and CS in functional independence (OR, 0.31; 95%CI, 0.10–0.97) and favorable outcome (OR, 0.24; 95%CI, 0.07–0.75).

**Conclusion:**

Our retrospective analysis suggested that the anesthesia strategy may affect outcome, in which general anesthesia may result in less favorable outcomes. Nevertheless, future multicenter randomized controlled trials are needed to confirm our findings.

## Background

Basilar artery occlusion (BAO) accounts for 1% of all strokes and 8% of symptomatic posterior circulation ischemia strokes [1], with a high mortality about 80% or more in patients treated conventionally [2]. Recently, with the development of noninvasive imaging with computed tomography and magnetic resonance techniques and invasive imaging with digital subtraction angiography, the diagnosis and treatment of BAO has greatly improved. Therefore, BAO has evolved from a nearly fatal disease to a treatable one [1]. The modern endovascular therapy (EVT) technique involving stent-retrievable mechanical thrombectomy has been validated in several large randomized controlled trials (RCTs) related to anterior circulation [3–6]. It is also effective and safe to use the same technique for BAO [7]. The recanalization rate has been improved by the use of stent retrievers, and approximately 80–90% of all blocked basilar arteries can be recanalized with current endovascular treatment [1]. However, despite these improvements in treatment, more than one in two patients still experience functional dependency [modified Rankin Scale (mRS) score > 2 after 3 months] [1, 8]. Clinical outcomes depend on patient-specific factors and procedure considerations [9]. Previous studies have suggested that anesthetic management during EVT may substantially affect outcomes of anterior circulation stroke (ACS) [10, 11].

Previous retrospective studies have suggested that general anesthesia (GA) may be associated with worse outcomes than conscious sedation (CS) for EVT [12, 13]. In contrast, some prospective studies have shown that outcomes were equivalent or better with GA than CS for anterior circulation thrombectomy [14–16]. However, posterior circulation stroke (PCS) is different from ACS in stroke etiology and outcome, because PCS is more often due to atherosclerosis [1], while ACS is more often classified as cardioembolic stroke [17]. The effect of anesthesia type of PCS on clinical outcomes seems to be different from ACS and the data on anesthesia used in patients with BAO is rare [9]. Consequently, the relationship between anesthesia and prognosis of patients with BAO after EVT is still uncertain.

To date, one study focused on posterior circulation stroke has been published, showing that monitored anesthesia care is as safe and effective as GA [9]. This study included all vertebrobasilar occlusion strokes. We therefore used a retrospective design to explore the effect of the type of anesthesia (GA and CS) on the outcomes of patients who received EVT in the setting of acute BAO.

## Methods

### Study population

This study involved a retrospective analysis examining the effect of the type of anesthesia on the outcomes of consecutive patients with BAO treated by EVT at the Beijing Tiantan Hospital. Patients admitted between January 2012 and July 2018 were included and those who required emergent intubation prior to EVT were excluded. All protocols were approved by the Institutional Review Board of Beijing Tiantan Hospital, and all patients or their relatives provided written consent for participation.

### Baseline data collection

Clinical baseline variables, including age, sex, vascular risk factors, mode of stroke onset, blood pressure, stroke severity as expressed by the National Institutes of Health Stroke Scale (NIHSS) score, laboratory tests, imaging modality, anatomical and morphological characteristics of BAO, stroke type according to the Trial of Org 10,172 in Acute Stroke Treatment (TOAST) classification [18], perioperative management, details of EVT, postsurgical modified Thrombolysis in Cerebral Infarction (mTICI) score [19], and clinical outcomes within 90 days, were prospectively collected. The time of BAO onset as described by the patients or witnesses was also prospectively recorded; if unknown, it was considered to be the last time the patient was found well. In patients with mild symptoms followed by a sudden onset of decreased consciousness, the time of deterioration of clinical status was estimated as the time of BAO onset.

### Anesthesia management

The anesthesia strategy was pre-designed based on the Practice Guidelines for Sedation and Analgesia by Non-Anesthesiologists, due to the continuum ranging of level of sedation from minimal sedation to GA [20]. The recommendation of anesthesia for patients was a joint decision between anesthesiologist and neurointerventionalist. Patients with clear consciousness and satisfactory airway reflex function are often recommended to undergo CS, while patients with poor preoperative consciousness and poor airway protective reflex are typically treated with general anesthesia. The target systolic blood pressure (SBP) before recanalization was maintained at 140-160 mmHg. Patients whose blood pressure was lower than this standard after sedation should undergo vasopressor medications to maintain blood pressure. For patients in the GA group, anesthesia was induced with sufentanil (0.2 μg/kg) or propofol (1–2 mg/kg). Muscle relaxation was achieved with rocuronium (0.6 mg/kg), followed by placement of a laryngeal mask or endotracheal intubation for providing mechanical ventilation to maintain end tidal carbon dioxide tension levels of 35–40 mmHg. Anesthesia was maintained with infusions of remifentanil at 0.1–0.2 μg/kg/min and propofol at 2–6 mg/kg/h. In the CS group, sedation was achieved with a low dose of propofol (2–4 mg/kg/h), and patients were given supplemental oxygen via a face mask. Anesthesiologists were involved in all procedures.

### Outcome measurement

The modified Rankin Scale (mRS) was used to evaluate functional outcomes at 90 days. Follow-up was conducted via telephone interview by trained assessors who were not involved in the study procedure. According to the Basilar Artery International Cooperation Study definition [21], functional independence was defined as mRS ≤2, and favorable outcome was defined as mRS ≤3. In this study, the clinical outcome measures were functional independence, favorable outcome and death at 90 days after the procedure.

### Statistical analysis

Study data were collected on standard forms, evaluated for completeness, and double keyed into an EpiData statistics data document. The baseline and outcome data were described using means (standard deviations) for normally distributed continuous variables or medians (25th and 75th percentiles) for non-normally distributed continuous and ordinal variables. Frequencies or proportions were used for categorical variables. Independent-samples t-test was used for normally distributed continuous variables, Mann-Whitney U test for non-normally distributed continuous and ordinal variables, while Pearson’s chi-square test or Fisher’s exact test was used to compare the frequencies or proportions between the CS and GA groups. For comparing the 90-day outcomes between both groups, odds ratios (ORs) with 95% confidence intervals (CIs) and adjusted ORs with 95% CIs were calculated using univariate and multivariable logistic regression models. To improve the reliability of the results, three multivariable regression models were used. In model 1, we adjusted only for age, sex, NIHSS, and median time of onset to puncture. In model 2, we additionally adjusted for the baseline variables with a difference at a level of *P* < 0.1 between both groups. In model 3, we further adjusted for the confounders selected on the basis of the change-in-estimate criterion, by which a variable can be included in the final model if its inclusion in the regression model [logit(Y) = β_0_ + β_1_ × X + β_2_ × Z, where X indicates the type of anesthesia, Y indicates the 90-day outcome, and Z indicates the included variable] produced a change in the regression coefficient of “X” by at least 10% compared with the one in the basic regression model [logit(Y) = β_0_ + β_1_ × X] [22]. Statistical significance was set at *P* < 0.05. All statistical analyses were performed with the statistical software package R (http://www.R-project.org, The R Foundation) and Empowerstats (http://www.empowerstats.com, X&Y Solutions, Inc., Boston, MA).

## Results

### Patient characteristics

**Fig. 1:**
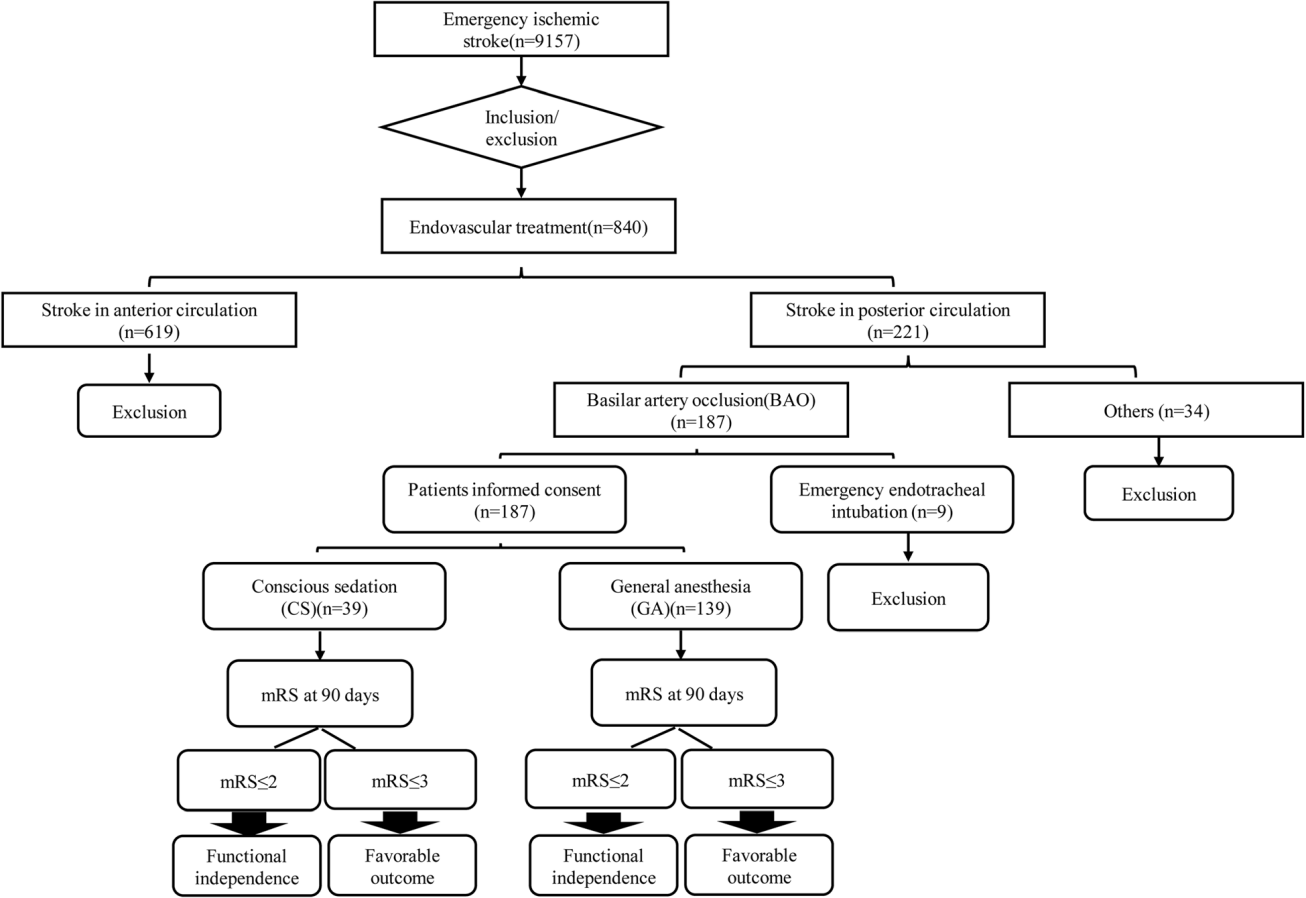
Flow chart of the study sample. BAO indicates basilar artery occlusion; CS, conscious Sedation; GA, general anesthesia; mRS, modified Rankin Scale

A total of 221 consecutive patients with PCS were treated with emergency EVT, including 187 patients with BAO. Nine patients with BAO who required emergent intubation prior to EVT were excluded in this analysis (Fig. 1). Their average age was 60 ± 10 years, 150 patients (84.3%) were men. The median admission NIHSS score was 20.5 [interquartile range (IQR) 10–33], 130 patients (73.0%) were treated by stent-retriever thrombectomy, the median time from onset to puncture was 7 (IQR 5–10) hours. In 151 cases (84.8%), patients were successfully recanalized (mTICI 2b-3). In all, 139 patients (78.1%) underwent GA and 39 patients (21.9%) underwent CS.

Baseline characteristics and procedural parameters are summarized in Table 1. Patients who underwent GA had higher NIHSS scores on admission [22 (IQR 11–35) versus 16 (IQR 6–30) points, *P* = 0.04], more often underwent thrombectomy using a stent retriever (79.1% versus 51.3%), more time needed to maintain anesthetic sedation [1.5 (IQR 1–2) versus 1 (IQR 1–2) hours, *P* = 0.01)], and more often received an infusion of tirofiban (73.4% versus 53.8%, *P* = 0.02). Compared the CS group, the GA group showed a different tendency with respect to sex distribution (87.1% versus 74.4%, *P* = 0.05), previous ischemic stroke (17.3% versus 30.8%, *P* = 0.07), blood glucose (8.6 mmol/L versus 9.8 mmol/L, *P* = 0.05), and mode of stroke onset (acute, 48.2% versus 53.8%; progressive, 48.9% versus 35.9%; and fluctuating, 2.9% versus 10.3%; *P* = 0.08). There were no significant differences between the groups in age (60 ± 10 years versus 60 ± 11 years, *P* = 0.96), onset to treatment time [7 (IQR 5–10) versus 7 (IQR 5–13) hours, *P* = 0.71], puncture to recanalization time [1 (IQR 0.5–2) versus 1 (IQR 0.5–2) hours, *P* = 0.34)], or successful recanalization (84.9% versus 84.6%, *P* = 0.97).

**Table 1:** Baseline characteristics of patients under GA and CS

Variable name	Overall (*n* = 178)	GA (*n* = 139)	CS (*n* = 39)	P
**Demographic data**				
Age, mean (SD), years	60 (10)	60 (10)	60 (11)	0.96
Male sex	150 (84.3)	121 (87.1)	29 (74.4)	0.05
**Vascular risk factors**				
Hypertension	125 (70.2)	99 (71.2)	26 (66.7)	0.58
Diabetes mellitus	50 (28.1)	41 (29.5)	9 (23.1)	0.43
Dyslipidemia	29 (16.3)	26 (18.7)	3 (7.7)	0.14
Coronary heart disease	21 (11.8)	16 (11.5)	5 (12.8)	0.78
Previous ischemic stroke	36 (20.2)	24 (17.3)	12 (30.8)	0.07
Current smoking	66 (37.1)	52(37.4)	14 (35.9)	0.86
**Clinical characteristics**				
Mode of stroke onset				0.08
Acute	88 (49.4)	67 (48.2)	21(53.8)	
Progressive	82 (46.1)	68 (48.9)	14 (35.9)	
Fluctuating	8 (4.5)	4 (2.9)	4 (10.3)	
Pre-procedural SBP, mean (SD),mmHg	160(25)	160(26)	160 (24)	0.86
Post-procedural SBP, mean (SD),mmHg	144 (21)	143 (22)	146 (19)	0.57
NIHSS score, median (IQR)	20.5 (10–33)	22 (11–35)	16(6–30)	0.04
mRS before stroke, median (IQR)	0 (0–0)	0 (0–0)	0 (0–0)	0.30
WBCs, mean (SD), ×1 0^9^	10.8 (3.8)	11.0 (3.7)	10.3 (4.0)	0.29
Blood glucose, mean (SD), mmol/L	8.8 (3.5)	8.6 (3.2)	9.8 (4.4)	0.05
Creatinine, mean (SD), umol/L	70.0 (21.7)	71.5 (20.7)	64.8 (24.7)	0.10
pc-ASPECTS on DWI, median (IQR)	6 (5–8)	7 (5–8)	6 (4–8)	0.81
PMI on DWI, median (IQR)	2 (0–4)	2 (0.25–4)	2 (0–3)	0.58
Occlusion site				0.99
Proximal BA	100 (56.2)	78 (56.1)	22 (56.4)	
Middle BA	51 (28.7)	40 (28.8)	11 (28.2)	
Distal BA	27 (15.2)	21 (15.1)	6 (15.4)	
Tandem lesion	24 (13.5)	17 (12.2)	7 (17.9)	0.36
Underlying ICAS	111 (62.4)	89 (64.0)	22 (56.4)	0.39
ASITN/SIR collateral system				0.68
Grade 0–1	76 (42.7)	61 (43.9)	15 (38.5)	
Grade 2	81 (45.5)	63 (45.3)	18 (46.2)	
Grade 3–4	21 (11.8)	15 (10.8)	6 (15.4)	
Stroke subtype by TOAST criteria				0.82
Large artery arteriosclerosis	143 (80.3)	112 (80.6)	31 (79.5)	
Cardioembolic	28 (15.7)	21 (15.1)	7 (17.9)	
Other or unknown etiology	7 (3.9)	6 (4.3)	1 (2.6)	
**Procedural features**				
Prior use of intravenous tPA	35 (19.7)	26 (18.7)	9 (23.1)	0.54
Use of Solitaire retriever	130 (73.0)	110 (79.1)	20 (51.3)	< 0.01
No. of passes, median (IQR)	1 (1–2)	1 (1–2)	1 (1–2)	0.44
Intra-arterial tPA or Urokinase	40(22.5)	29 (20.9)	11 (28.2)	0.33
Infusion of Tirofiban	123 (69.1)	102 (73.4)	21 (53.8)	0.02
Heparinization	76 (42.7)	60 (43.2)	16 (41.0)	0.81
Angioplasty				< 0.01
No	57 (32.0)	36 (25.9)	21 (53.8)	
Balloon alone	31 (17.4)	25 (18.0)	6 (15.4)	
stenting	90 (50.6)	78 (56.1)	12 (30.8)	
OTP, median (IQR), hours	7 (5–10)	7 (5–10)	7 (5–13)	0.71
PTR, median (IQR), hours	1(0.5–2)	1(0.5–2)	1(0.5–2)	0.34
Anesthesia time (IQR), hours	1.5 (1–2)	1.5 (1–2)	1 (1–2)	0.01
Successful recanaliation (mTICI 2b-3)	151 (84.8)	118 (84.9)	33 (84.6)	0.97

Values are numbers with percentages in parentheses, unless indicated otherwise

*GA* indicates general anesthesia, *CS* conscious sedation, *SD* standard deviation, *SBP* systolic blood pressure, *NIHSS* National Institutes of Health Stroke Scale, *mRS* modified Rankin Scale, *WBC* white blood cell, *IQR* interquartile range, *pc-ASPECTS* posterior circulation Acute Stroke Prognosis Early CT Score, *DWI* diffusion weighted imaging, *PMI* Pons-Midbrain Index, *BA* basilar artery, *ICAS* intracranial atherosclerotic stenosis, *ASITN/SIR* American Society of Interventional and Therapeutic Neuroradiology/Society of Interventional Radiology, *TOAST* Trial of Org 10,172 in Acute Stroke Treatment, *tPA* tissue plasminogen activator, *OTP* onset-to-puncture time, *PTR* puncture to recanalization time, *mTICI* modified Thrombolysis In Cerebral Infarction scale

### Association of anesthesia with 90-day outcomes in univariate analysis

**Fig. 2:**
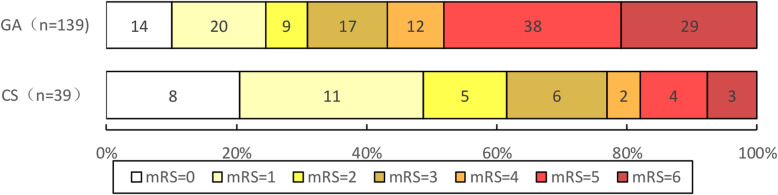
The Distribution of mRS at 90-day stratified by GA and CS. CS indicates conscious Sedation; GA, general anesthesia; mRS, modified Rankin Scale

The functional and favorable outcomes and mortality at 90 days are given in Table 2 and the distribution of mRS at 90-day is presented in Fig. 2. A total of 43 patients (30.9%) who underwent GA and 24 patients (61.5%) who underwent CS had functional independence (mRS 0–2). A favorable outcome (mRS 0–3) was seen in 60 patients (43.2%) who underwent GA and in 30 patients (76.9%) who underwent CS. At 90 days, 29 patients (20.9%) who underwent GA and 3 patients (7.7%) who underwent CS had died. In univariate analysis, GA was associated with less functional independence (OR, 0.28; 95% CI, 0.13–0.59; *P* < 0.01) and less favorable outcome (OR, 0.23; 95% CI, 0.10–0.52; *P* < 0.01) than was CS. There was no significant difference in mortality between the GA and CS group (*P* = 0.07).

**Table 2:** Comparison of 90-day outcome between patients under GA and CS

Outcome variable	GA	CS	Univariate analysis		Multivariable analysis					
					Model 1		Model 2		Model 3	
			cOR (95% CI)	*P* value	aOR (95% CI)	*P* value	aOR (95% CI)	*P* value	aOR (95% CI)	*P* value
Functional independence (mRS 0–2) at 90 days	43/139 (30.9)	24/39 (61.5)	0.28(0.13–0.59)	< 0.01	0.31(0.13–0.75)	0.01	0.29 (0.09–0.97)	0.045	0.31(0.10–0.97)	0.045 *
Favorable outcome (mRS 0–3) at 90 days	60/139 (43.2)	30/39 (76.9)	0.23(0.10–0.52)	< 0.01	0.26(0.10–0.64)	< 0.01	0.25 (0.07–0.83)	0.02	0.24(0.07–0.75)	0.02 †
Death at 90 days	29/139 (20.9)	3/39 (7.7)	3.16(0.91–11.01)	0.07	2.57(0.70–9.52)	0.16	3.36 (0.48–23.68)	0.22	2.99(0.46–19.74)	0.25 ‡

Model 1 adjusted for age, sex, from vascular occlusion to puncture, NIHSS score

Model 2 adjusted for age, sex, from vascular occlusion to puncture, NIHSS score, previous ischemic stroke, Mode of stroke onset, Blood glucose, Use of Solitaire retriever, Infusion of Tirofiban, Angioplasty

*GA* indicates general anesthesia, *CS* conscious sedation, *cOR* crude odds ratio, *aOR* adjusted odds ratio, *CI* confidence interval, *mRS* modified Rankin Scale

*Adjusted for age, sex, from vascular occlusion to puncture, NIHSS score, previous ischemic stroke, angioplasty, anesthesia time

†Adjusted for age, sex, from vascular occlusion to puncture,NIHSS score, use solitaire retriever, blood glucose, anesthesia time

‡Adjusted for age, sex, from vascular occlusion to puncture, NIHSS score, previous ischemic stroke, blood glucose, use of solitaire, angioplasty, anesthesia time

### Association of anesthesia with 90-day outcomes in multivariable analysis

In model 1, there were significant differences between GA and CS in functional independence (OR, 0.31; 95% CI, 0.13–0.75; *P* = 0.01) and favorable outcome (OR, 0.26; 95% CI, 0.10–0.64; *P* < 0.01). There was no significant difference in mortality between GA and CS (*P* = 0.16). In model 2, patients who underwent GA had less functional independence (OR, 0.29; 95%CI, 0.09–0.97; *P* = 0.045) and less favorable outcomes (OR, 0.25; 95%CI, 0.07–0.83; *P* = 0.02) than those who underwent CS. There was no significant difference in mortality between GA and CS (*P* = 0.22). In model 3, GA was still associated with less functional independence (OR, 0.31; 95% CI, 0.10–0.97; *P* = 0.045) and a less favorable outcome (OR, 0.24; 95% CI, 0.07–0.75; *P* = 0.02) than was CS. There was no significant difference in mortality between GA and CS (*P* = 0.25).

## Discussion

Many patients with acute BAO need intubation and ventilation, analgesia-based sedation. Some neurointerventionalists prefer GA during EVT because patients can remain motionless for safer intracranial navigation of devices and better imaging. However, a consensus about the effect of anesthesia has not been reached. Therefore, we hoped to explore this critical issue in this study. We found that functional independence (mRS 0–2) and favorable outcomes (mRS 0–3) at 90 days after the EVT were significantly different between the patients who underwent GA and those who underwent CS, but mortality did not differ between the two groups. Patients treated with GA may have a worse clinical prognosis after EVT in the setting of acute BAO than those treated with CS.

In our study, GA was in form of induction + airway + a slightly higher dose of propofol, and propofol was used to maintain anesthesia, without using gas inhalation, while a low-dose of propofol was used for CS. This anesthetic strategy was similar to two other anterior circulation-based trials (SIESTA and GOLIATH) [16, 23]. Previous studies have shown that propofol can reduce cerebral blood flow (CBF) and cerebral metabolic rate of oxygen in a dose-dependent manner [24, 25]. However, no enough clinical evidence has been reported to support the use of any anesthetic agents in GA [15, 16, 23]. Moreover, the PCS, especially BAO, mostly involves the brainstem. Harbouring numerous vital nucleus such as respiratory, heartbeat and blood pressure control centers, where the collateral circulation of blood flow exists anterior circulation reflux, but it is still relatively fragile. In our study, we found that BAO patients treated with GA was associated with worse clinical outcomes than those treated with CS. Thereby, we proposed that we should pay more attention to anesthesia strategy and the control of deep anesthesia while actively carrying out mechanical endovascular therapy.

In addition, the results in the present study are consistent with those of other retrospective studies that have found inferior neurological outcomes with GA as opposed to CS for patients presenting with anterior circulation strokes [12, 13, 26]. Berkhemer et al. [13] performed a post hoc analysis of the use of CS in the endovascular treatment of the MRCLEAN trial, and found a worse clinical outcome in those patients who underwent GA compared with patients who underwent CS. This may be because of higher stroke severity, hypotension, or hypocapnia during the procedure under GA, causing a poor prognosis. Studies have shown that hypotension may enhance risk to the ischemic penumbra and hypocapnia potentially leading to brain tissue hypoxia in the ischemic penumbra [27, 28]. Other prospective studies, such as the ANSTROKE study [15], showed no difference between the two anesthetic techniques in neurological outcome 3 months after stroke. Similarly, the SIESTA [16] and GOLIATH [14] studies demonstrated no advantage in the use of CS over GA. The different findings between the prospective studies and the retrospective studies may expose the problem of confounders in terms of the different designs. Many confounders, such as the basic characteristics of patients, onset to treatment time, effect of anesthetic factors, the sites of occlusion, and procedural features, may lead to inconsistencies.

However, most studies have limited enrollment to patients with acute anterior circulation occlusions, and only few studies have focused on the anesthesia for EVT for acute stroke in the posterior circulation. Jadhav et al. [9]. in their study indicated that monitored anesthesia care was safe and effective in EVT for acute vertebrobasilar occlusion strokes. They lacked the imaging data of patients and assessment of collateral circulation, which might affect the results. There are several limitations to our study. First, because our study was a single-center retrospective cohort study, and not an RCT, there may be a selection bias between the two groups, especially in patients with GA with high NIHSS scores. However, we used three multivariable regression models, in which the NIHSS score was adjusted as a confounding factor. Besides, a randomized controlled study, namely the “Choice of Anesthesia for Endovascular Treatment of Acute Ischemic Stroke at Posterior Circulation: Protocol for an Exploratory Randomized Controlled Study [29]” was already in progress. Second, we did not investigate periprocedural blood pressure fluctuations, changes in blood oxygen concentrations, or complications such as rates of aspiration pneumonia and subsequent intubation, which may have influenced our results. Although adjusted by models, potential confounders that we did not or could not measure may have influenced the outcome. Third, the sample size was relatively limited, especially the number of patients with CS. Any try of matching correction or propensity score matching (PSM) between the GA and CS would further reduce the sample size, but would bring statistical bias. Thereby, three multivariable logistic regression models rather than matching correction or PSM were used to adjust NIHSS score. In future research, we needed to further expand the sample size. Given these limitations, our results should be carefully interpreted.

## Conclusion

In our study, GA for endovascular intervention in patients with acute BAO was associated with worse neurological outcomes than those associated with CS, but GA did not increase mortality. This suggests that anesthesia may affect the prognosis of patients with acute BAO who undergo EVT. Future multicenter RCTs are needed to determine which type of anesthesia may be better for patients with acute BAO undergoing EVT.

## Data Availability

The datasets generated during and/or analysed during the current study are available from the corresponding author on reasonable request.

## Abbreviations

ACS: Anterior circulation stroke
BAO: Basilar artery occlusion
CI: Confidence interval
CS: Conscious sedation
EVT: Endovascular therapy
GA: General anesthesia
IQR: Interquartile range
mRS: Modified Rankin Scale
mTICI: Modified Thrombolysis in Cerebral Infarction
NIHSS: National Institutes of Health Stroke Scale
OR: Odds ratio
PCS: Posterior circulation stroke
PSM: Propensity score matching
RCTs: Randomized controlled trials
SBP: Systolic blood pressure
TOAST: Trial of Org 10,172 in Acute Stroke Treatment
